# Knowledge, Attitudes and Practice of Condom Use among Males Aged (15-49) Years in Erbil Governorate

**DOI:** 10.5539/gjhs.v4n4p27

**Published:** 2012-05-21

**Authors:** Aziz Sulaiman Ismael, Jwan M. Sabir Zangana

**Affiliations:** 1 Specialist of Family Medicine in Brayatee family center, Erbil, Iraq; 2 Lecturer in Community Medicine Department, College of Medicine, Hawler Medical University, Erbil, Iraq

**Keywords:** condom, knowledge, sexual transmitted infection (STIs), attitude, perception

## Abstract

**Background and Objectives:**

Globally, condom is an important method of family planning and prevention of sexually transmitted infections especially human immune deficiency virus HIV/acquired immune deficiency syndrome AIDS. Family planning saves lives of women and children and improves the quality of life. This study was conducted to assess knowledge, attitudes and practices in addition to socio-demographic factors of condom use among males in Erbil governorate.

**Subjects and method:**

A cross sectional study conducted on randomly selected sample of 600 males aged 15-49 years from 3 districts of Erbil governorate of Iraqi Kurdistan region by using multistage cluster sampling method to assess their knowledge, attitudes and practice of condom use.

**Results:**

Only 12% of respondents had ever used condoms. The main reason for condom use was for family planning in about 91.7%. About a quarter of respondents reported knowing how to use condom correctly. Condoms were considered by respondents as an effective method of contraception and prevention of sexually transmitted infections 33.2% and 28.3% respectively. While 30.3% of them believed that condom use had some harmful effects. The main reason to non condom use was lack of need in 45.5%, fertility related reasons in 17% and the use of other methods by the female partner 13.6%. Although 64% of respondents heard about AIDS /HIV and 71.7% about STIS in general, only few felt that they are at risk of STIs 9.5% and HIV infection 8.5%.

**Conclusion:**

The study showed that the rate of condom use was low among the studied sample. This was due to lack of knowledge about proper and effective use of condoms, low perception of risk of HIV and other STIs, misperception about harmful effects of condoms and the use of other family planning methods by respondents and their female sexual partner.

## 1. Introduction

Condom is one of the most popular forms of mechanical barriers as it provides protection for the genital tract from sexually transmitted infections (STIs). It also prevents pregnancy by acting as a barrier stopping semen from passing into the vagina (Jain et al., 2009).

The use of the condoms was traced back to several thousand years ago. Condoms were invented in the fifteenth century in response to syphilis epidemic in Europe. Since then, the texture of condoms was developed from different kinds of materials such as leather and animal gut. During eighteenth century, the technological development improved the quality of condoms. Rubber was developed as material because of its strength and elasticity. The role of male condom for both contraception and prevention of STIs was established in Europe during this century (Lewis, 2000).

Worldwide condom use during sexual intercourse, an estimated 44 million couples use condom for family planning while as many as 60% of all condoms are used outside marriage (Gardner et al., 2001).

The effectiveness of condoms in preventing pregnancy or STIs depends on the user. Previous studies showed that pregnancy rate among correct condom users is about 2% per year. The risk of pregnancy or STI is greater when condoms are not used correctly and consistently with every sexual act. However, when its used every time and in the approved manner it could prevent up to 80 to 95% of HIV transmission (USAIDS, WHO, 2007). Condoms also reduce the risk of STIs spread by skin to skin contact, such as herpes and Human Papilloma virus.

In Egypt, results of a study on the knowledge, attitude of condom use in 2007 showed that although condoms was considered effective method of contraception and prevention of STIs by 60%, only 23% had ever used condoms solely for contraception (Kabbash, 2007).

Similar study was conducted in Rajshahi district of Bangladesh which showed that knowledge and use of contraception were low. Condoms were only use in 17% of the cases (Moisur et al., 2009). Another study conducted in Pakistan, showed low knowledge regarding the appropriate use of condoms even among contraceptive users (Fikree et al., 2005).

Family planning save lives of women and children and improves quality of life (World Health Organization, 1995). Men seem to play a powerful role in the reproductive decisions; their actions can have unhealthy and even dangerous outcomes on family planning (Dernnan, 1998).

Likewise, A study which was conducted on family planning in Baghdad capital of Iraq in 2003 showed that knowledge about condoms were patchy and although males knew that there are some benefits in using condoms for family planning 84% only 4.2% considered using this means of contraception.

### 1.1 Rational

No similar study has been done in Kurdistan of Iraq especially in Erbil city regarding knowledge, attitude, and practice of condom use among male aged 15-49 years in Erbil governorate.

### 1.2 Aim

To find out the proportion of condom users among males and to study their knowledge, attitude, and practicing it as family planning mean or as preventive measure against sexual transmitted infections.

## 2. Subject and Methods

A cross sectional study conducted in 15 randomly selected primary health care centers (PHCs) which belongs to three randomly selected districts of Erbil governorate of Iraqi Kurdistan region (city center, Erbil periphery and shaqlqwa) from 1^st^ of March 2010 to 1^st^ of March 2011. The target population was adult males aged 15-49 years which are chosen from different educational and occupational groups. A verbal consent was taken from each recruited respondent. They were informed that participation in the study was voluntary. Permission was obtained from Directorate of Health and from four teaching hospitals in Erbil city to carry out the study.

The sample size was calculated by EPI info-6 Jan. 2001. Computer program issued by the center for disease control (CDC) and world health organization (WHO), the following data were entered in order to calculate the sample size.

Estimated Erbil population is around 1,571,745. Estimated prevalence (expected frequency) of condom use by men in region is approximately 10 % and worst acceptable value (precision) of 12.5 %. A representative sample size of 553 was selected. For convenience, a sample of 600 respondents was recruited.

Closed ended self administered questionnaire was used by researchers and reviewed by the supervisor. The questionnaire included information on socio-demographic details which includes name, age, residence, marital status, income, occupation (driver and non driver due to high incidence of condom use among such job), years of formal education and crowding index, passion of car, type of house, number of living children, educational state with occupation of their wives with their knowledge and attitudes toward condom use, patterns of condom use and barriers to use, respondents knowledge about HIV/AIDS and other (STIs).

### 2.1 Statistical Analysis

Questionnaires were coded after arrangement of information. Data were entered and analyzed using statistical packages for social sciences (SPSS, version 18). Two approaches were used; descriptive and analytic. The descriptive approach included calculation of the frequencies, percentages, mean SD.s and for the second approach; Chi-square test and Fisher’s exact test were used to test the association between categorical variables.

## 3. Results

### 3.1 Socio-Demographic Characteristics of the Sample

Overall 600 respondents aged 15-49 years old from Erbil governorate were recruited for this study. The respondents were distributed according to their age, district location, place of residence, marital status, occupation and educational level of the respondents and their wives, number of living children, crowding index, possession of car and type of housing as shown in Table 1 & 2.

**Table 1:** Distribution of sample by selected demographic factors

Variables	No.	%
Place of residence		
Urban	540	90
Rural	60	10
Respondent occupation		
Driver	132	22
*Non-driver	468	78
Marital status		
Single	192	32
Married	384	64
Divorced	9	1.5
Widowed	15	2.5
Wife occupation		
Non- employed (Housewives)	267	69.5
Employed	117	30.5
No. of living children		
0-1	58	14.2
2-4	254	62.3
≥ 5	96	23.5

* Non-drivers included government employee, self employed and students

**Table 2:** Distribution of sample by selected socioeconomic factor

Variables	No.	%
Respondent educational level		
Illiterate	71	11.8
Read and Write	74	12.3
Primary	119	19.8
Secondary	292	48.7
Diploma, university and higher education	44	7.3
Wife educational level		
Illiterate	93	24.3
Read and Write	64	16.6
Primary	74	19.3
Secondary	131	34.2
Diploma, university and higher education	22	5.7
Crowding Index		
< 1.5	147	24.5
1.5 – 2.9	361	60.2
≥ 3	92	15.3
Possession of Car		
Yes	295	50.8
No	305	49.2
Type of Housing		
Owned	336	56
Partially Owned	64	10.7
Rented	200	33.3
Total	600	100

### 3.2 Respondent Knowledge and Attitudes toward Condom Use

Table 3 shows that 33.2% of respondents considered condom as an effective method of contraception, while 28.3% of them considered condoms as an effective method of prevention of (STIs). Only 25.8% of the respondents reported to have enough knowledge about proper condom use; and 30.2% thought that condom use may have some harmful effects.

**Table 3:** Distribution of sample by knowledge & attitudes toward condom use

Knowledge and attitudes toward condom use	Yes	%	No	%
Condoms are effective method of contraception	199	33.2	401	66.8
Condoms are effective for prevention of STIs	170	28.3	430	71.7
Had enough knowledge about proper condom use	155	25.8	445	74.2
Need more information about proper use of condoms	430	71.7	170	28.3
Condoms use has some harmful effects	181	30.2	419	69.8
Feel embarrassed when buying condom	64	10.7	536	89.3
Condoms are available in the neighborhood	513	85.5	87	14.5
Might use condom in the future	155	29.4	375	70.6

### 3.3 Condom Practicing

Table 4 shows that the rate of condom use among 600 respondents was 12%, while 88% never used condoms in their life. The main reason for condom use among 12% of condom users was for family planning 91.7% and 2.8% used condom for prevention of STIs.

**Table 4:** Distribution of sample by condom practicing

Condom practicing	No.	%
Condom user	72	12
Non-condom user	528	88
Total	600	100
Reasons for condom use		
Family planning	66	91.7
Prevention STIs	2	2.8
Both	4	5.6
Total	72	100

Table 5 shows the reasons for non-condom use, includes: refuse condom use by 44.5% of respondents (being single or married), 17.8% respondents reported desire for conception, using other family methods by their wife and respondents, decrease sexual pleasure, rejection by partner and religious reason were also reported.

**Table 5:** Distribution of sample by reasons for non-condom use

Reasons for non-condom use	No.	%
Refuse condom use	235	44.5
Desire for conception	94	17.8
Use of other methods by wife	90	17
Use of coitus interrupts	72	13.6
Rejection by partner	13	2.5
Decrease sexual pleasure	13	2.5
Religious reason	11	2.1
Total	528	100

### 3.4 Associations between Socio-demographic Variables and Condom Use Respondent Age

Table 6 shows that among 12% condom users of different age groups, the highest level of condom use was among respondents of age group of 35-39 years, which was significantly higher than other age group (*x*^2^ =16.643, P = 0.005). The rate of condom use among age group 25-29 years was relatively high, but the difference from other age group was not significant. For age group 35-39 has a statistically significant association with the condom use.

**Table 6:** Association of respondent age groups and condom use *x*^2^

Age groups	Condom user		Non-condom user		Total		P-value

	No.	%	No.	%	No.	%	
15-19 years	1	2.5	39	97.5	40	100	0.07*
20-24 years	13	10.3	113	89.7	126	100	0.51
25-29 years	26	15.6	141	84.4	167	100	0.9
30-34 years	11	11.5	85	88.5	96	100	0.85
35- 39 years	15	22.4	52	77.6	67	100	0.005
40-44 years	5	6.6	71	93.4	76	100	0.12
45-49 years	1	3.6	27	96.4	28	100	0.23*
Total	72	12	528	88	600	100	

* Fischer’s’ exact test, P-value < 0.05 was significant

### 3.5 Place of Residence

Table 7 shows that among 12% condom users, 13% were living in urban, 3.3% were living in rural areas. The place of residence of respondents has a statistically significant association with condom use (*x*^2^ = 4.742, p= 0.016).

**Table 7:** Association of respondent place of residence and condom use

Residence	Condom user		Non condom user		Total		*χ^2^*	P-value

	No.	%	No.	%	No.	%		
Urban	70	13	470	87	540	100	4.742	0.016
Rural	2	3.3	58	96.7	60	100		
Total	72	12	528	88	600	100		

### 3.6 Respondent, Wife Educational Level

Table 8 shows among (12%) condom users, the lowest percentage of condom use were among illiterate, while the highest percentage of condom were among respondents with diploma, university and high education (*x*^2^ = 26.440, p<0.001). At the same time there was statistical significant association between condom user and educational state of their wives (*x*^2^ = 26.424, p<0.001).

**Table 8:** Association of respondent, wife educational level and condom use

Respondent educational level	Condom user		non condom user		Total		*χ^2^*	P-value

	No.	%	No.	%	No.	%		
Illiterate	3	4.2	68	95.8	71	100	26.440	<0.001
Read and write	6	8.1	68	91.9	74	100		
Primary	11	9.2	108	90.8	119	100		
Secondary school	37	12.7	255	87.3	292	100		
Diploma, university and HE	15	34.1	29	65.9	44	100		
Total	72	12	528	88	600	100		

Wife educational level	Condom user		non condom user		Total		*χ^2^*	P-value

	No.	%	No.	%	No.	%		
Illiterate	7	7.5	86	92.5	93	100	26.424	<0.001
Read and write	6	9.4	58	90.6	64	100		
Primary	12	16.2	62	83.8	74	100		
Secondary school	33	25.2	98	74.8	131	100		
Diploma, university and HE	10	45.5	12	54.5	22	100		
Total	66	19.89	316	80.11	384	100		

### 3.7 Socioeconomic Status, Marital Status and Wife Occupation

Table 9 shows that the highest level of condom use was among higher socioeconomic status, married and among respondents with employed spouse with statistical significant association of previous variable with condom.

**Table 9:** Association of socioeconomic status, marital status, wife occupation and condom use

Socio-economic status	Condom user		Non condom user		Total		*χ^2^*	P-value

	No.	%	No.	%	No.	%		
Low	3	3.4	86	96.6	89	100	20.772	0.001
Medium	32	9.6	300	90.4	332	100		
High	37	20.7	142	79.3	179	100		
Total	72	12	528	88	600	100		

Marital status	Condom user		Non condom user		Total		*χ^2^*	P-value

	No.	%	No.	%	No.	%		
Single	2	1	190	99	192	100	34.094	<0.001
Married	68	17.7	316	82.3	384	100		
Divorced	1	11.1	8	88.9	9	100		
Widowed	1	6.7	14	93.3	15	100		
Total	72	12	528	88	600	100		

Wife occupation	Condom user		Non condom user		Total		*χ^2^*	P-value

	No.	%	No.	%	No.	%		
Housewife	33	12.4	234	87.6	267	100	17. 204	<0.001
Employed	35	29.5	82	70.5	117	100		
Total	68*	316	82.3	17.7	384	100		

### 3.8 Respondent Occupation, No. Living of Children

Table 10 shows that there was no statistically significant association between respondent occupation, no. of living children and condom use.

**Table 10:** Association respondent occupation, no. of living children and condom use

No. of living children	Condom user		Non condom user		Total		*χ^2^*	P-value

	No.	%	No.	%	No.	%		
0-1	14	24.1	44	75.9	58	100	28.827	0.243
2-4	44	17.3	210	82.7	254	100		
≥ 5	13	13.5	83	86.5	96	100		
Total	72	12	528	88	408	100		

resprondent occupation	Condom user		Non-condomuser		Total		*χ^2^*	P-value

	No.	%	No.	%	No.	%		
Driver	21	15.9	111	84.1	132	100	5.373	0.112
*Non-drivers	51	10.89	417	89.11	468	100		
Total	72	12	528	88	600	100		

* Non-drivers included government employee, self employed and students

### 3.9 Association of Condom Use with Respondent Perception of HIV, STIs Risks

Table 11 shows that among (12%) condom users, the highest level of condom use was among respondents who perceived risk of HIV, STIs while the lowest level of condom use was among respondents who did not perceive risk of HIV and STIs.

**Table 11:** Association of respondent perceive risk of HIV, STIs and condom use

Perceived risk of HIV	Condom use		Condom non-use		Total		*χ^2^*	P- value

	No.	%	No.	%	No.	%		
No	37	6.7	512	93.3	549	100	169.254	<0.001
Yes	35	68.6	16	31.4	51	100		
Total	72	12	528	88	600	100		

Perceive risk of STIs	Condom user		Non condom user		Total		*χ^2^*	P- value

	No.	%	No.	%	No.	%		
No	36	6.6	507	93.4	543	100	156.095	<0.001
Yes	36	63.2	21	36.8	57	100		
Total	72	12	528	88	600	100		

## 4. Discussion

It is essential to study the pattern of condom use which is now not only important for family planning and reducing fertility indices but also a life saver by preventing HIV infection. When properly used, male condoms represent a proven and effective mean for family planning and preventing transmission of HIV/AIDS and other STIs (UNAIDS technical update. Geneva, UNAIDS, 2000). Men play a powerful and even dominant role in the reproductive decisions sometimes regardless of their partner wishes or health. Therefore, it is important to direct the focus of health programs to advocate for a healthy male sexual behavior (Lasse, 1997).

Among all respondents, condoms were considered as an effective method of contraception and prevention of transmission of sexually transmitted infections (STIs) (33.2%) (28.3%) respectively. The analysis of this study showed that the knowledge, attitude of condom use were low. Although condoms were reported by the majority of respondents 85.5% to be easily available, only 12% had ever used them, while 29.4% claimed that they might use them in the future. The low level of condom use in this study may be related to the observation that only 25.8% of the respondents reported having enough information about proper condom use and 71.7% required more information. The main obstacles for condom use was refusal of use by 44.5% by being single, having no sexual relationship or having monogamous relationship, desire for conception 17.8%, using other methods during marriage (use other methods by female partner or coitus interrupts), 17% 13.6% respectively, and perceived harmful effects of condom 30.2% as shown in Table 3.

This study showed that only 12% of respondents were condoms users. The main reason for condom use was for family planning 91.7%, 2.8% for prevention of STIs and (5.6%) for both family planning and prevention of STIs. There was a significant association between condom use and respondents age group by having a higher rate of use among 35-39 years age group. This result was consistent with a study conducted in Turkey (Ozvaris et al., 1998). This may be due to fact that younger age groups are more sexually active and by using condoms they could avoid unwanted pregnancy and eliminate STD transmission.

There was significant association between higher condom uses and being residence of urban areas, this result was in agreement with results of study conducted in Tanzania (Kapiga et al., 2003). Condoms are more accessible in those areas and there are a higher percentage of educated families with positive attitude toward condom use.

There was significant association between both of educational and marital state of respondent with condom use; this result is in agreement with a study conducted in Thailand and Mexico City respectively (Aekusuk, 2007, Hernandez- Giron et al., 1999).

There was higher level of condom use 20.7% among higher socioeconomic status, this results was consistent with a study conducted in India (Kamal & Huda, 2009). The result may be due to a better accessibility to family planning services and an easier access to information about awareness and prevention from HIV/AIDS and other STDs by people from higher socio-economic class.

Regarding the occupation of respondents, there was none significant association between occupation and condom use which was compatible with results of a study conducted in Thailand (Guaykie tikul et al., 1994).

Perceived risk of HIV and sexually transmitted infections was significantly associated with condom use as the highest level of use was among respondents who perceive a reduced risk of HIV and STIs (68.6%, 63.2%) transmission by condom use, respectively. This result was compatible with result of study done in (Bali) in Indonesia and in Japan, which revealed a statistical association between condom use and perceive risk of HIV and STIs (Ford et al., 2002; Amazaki & 1Shimizu, 2008).

## 5. Conclusion

The rate of condom use was low among studied sample, the respondent level of knowledge about proper and effective use of condoms for family planning and protection from STIs were low. The respondent level of awareness about HIV and other STIs among the studied sample was low in addition to misperceptions about harmful effects of condoms among studied sample. The main predictors of male condom use in this study were being married and perceived high risk of HIV & STIs transmission. The rate of male participation in family planning was low among studied sample. The highest levels of condom use were observed among high socioeconomic status, married, and high educational level couples, and urban residents.

## Limitation of the Study

Difficulties in accessing information about condom use due to cultural and social limitations require further anaylsis and research. In addition, the result of the study could not be generalized entirely to our population, hence, further studies needs to be carried out.
